# A case of penile fracture with complete urethral disruption during sexual intercourse: a case report

**DOI:** 10.1186/1752-1947-1-14

**Published:** 2007-05-02

**Authors:** Klemen Jagodič, Marko Erklavec, Igor Bizjak, Sandi Poteko, Helena Korošec Jagodič

**Affiliations:** 1 Department of Urology, General Hospital Celje, Slovenia; 2 Department of Anesthesiology, General Hospital Celje, Slovenia

## Abstract

Penile fracture is a rare condition. Primarily it is a rupture of the corpus cavernosum that occurs when the penis is erect. The rupture can also affect the corpus spongiosum and the urethra.

We report a case of a 37 year old man who presented with acute penile pain, penile swelling and the inability to pass urine after a blunt trauma during sexual intercourse. In emergency surgery we found bilateral partial rupture of the corpus cavernosum with complete urethral and corpus spongiosum disruption. In the one year follow up the patient presented with normal erectile and voiding function.

Emergency surgical repair in penile fracture can preserve erectile and voiding function.

## Background

Fracture of the penis is a relatively uncommon form of urologic trauma. It is a disruption of the tunica albuginea of one or both corpus cavernosum due to blunt trauma to the erect penis [1]. It can be accompanied by partial or complete urethral rupture or by injury of the dorsal nerve and vessels [2].

Tunica albuginea is one of the strongest fascia in the human body. One reason for the increased risk of penile fracture is that the tunica albuginea stretches and thins significantly during erection: in the flaccid state it is up to 2.4 mm thick; during erection it becomes as thin as 0.25 to 0.5 mm. Bitsch et al. and De Rose et al. proposed that an intracorporal pressure of 1500 mmHg or more during erection can tear the tunica albuginea [3,4].

The classic, "text – book" history of penile fracture is: a sudden cracking sound as the tunica tears followed by pain, rapid detumescence, swelling and discoloration of the penis with or without voiding problems [5].

## Case report

A 37 year old man presented with a sudden cracking sound and acute pain during sexual intercourse followed by rapid detumescence, penile swelling and discoloration. Pain was aggravated by trying to urinate, but he could not pass the urine. Six hours after the penile trauma the patient was admitted to the emergency department.

Physical examination revealed a swollen, ecchymotic penis, blood on the urethral meatus and palpably full bladder. A retrograde urethrogram showed complete disruption at the proximal third of the urethra. The patient underwent immediate surgical exploration and repair of the fracture.

**Figure 1:**
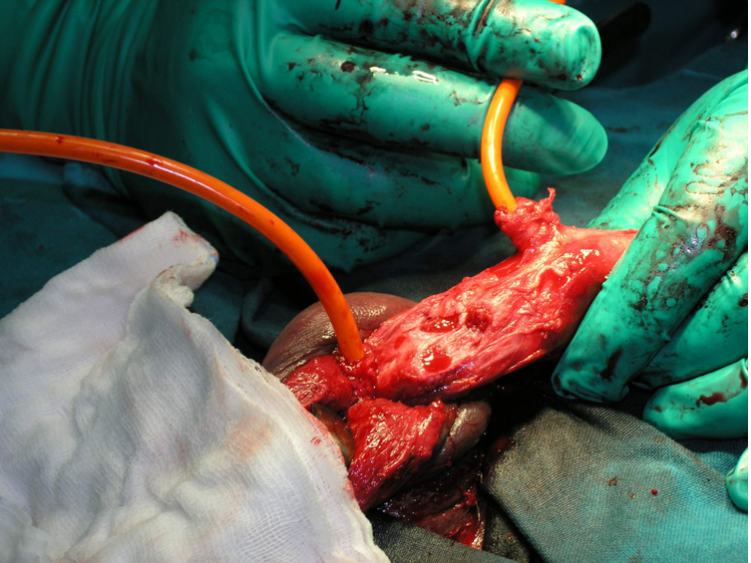
Complete urethral disruption with partial rupture of both corpus cavernosum.

**Figure 2:**
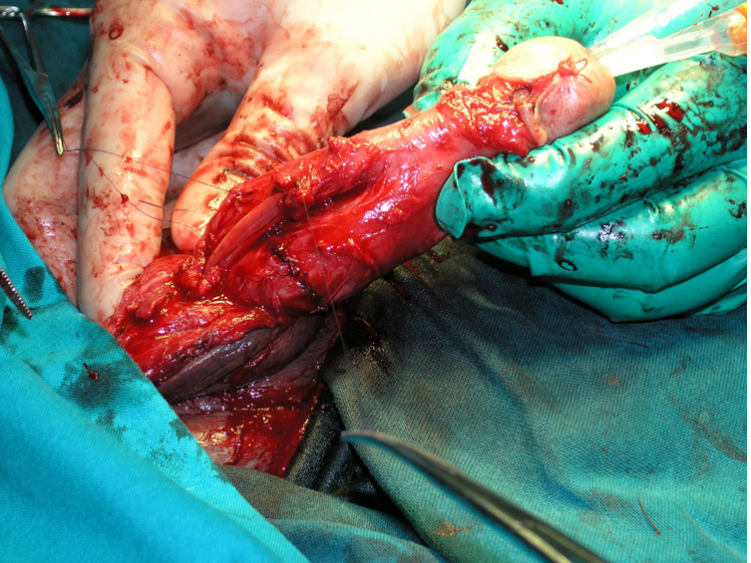
Sutured both corpus cavernosum and indwelled Foley catheter.

**Figure 3:**
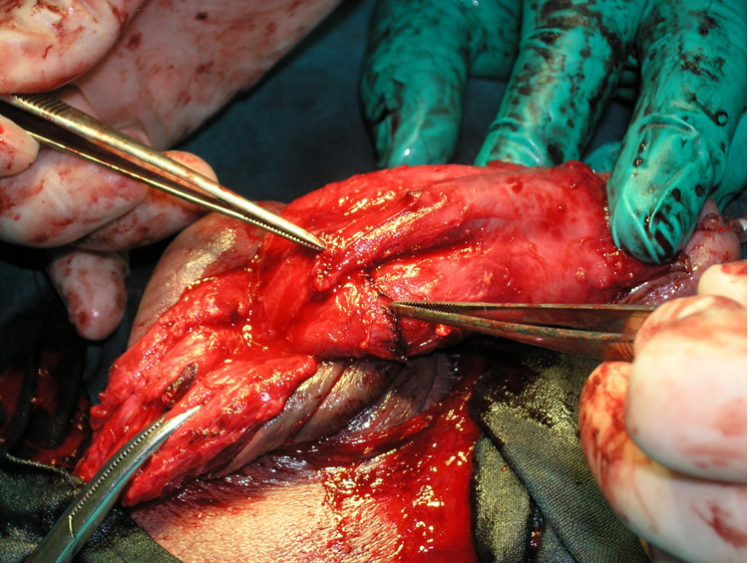
T-T anastomosis of the urethra.

Circumferential subcoronal degloving incision (circumcision – like) and hematoma evacuation presented a partial tear of the tunica albuginea of both corpus cavernosum and complete urethral disruption. After minimal debridement and mobilization of proximal and distal corpus spongiosum the urethra was spatulated (figure 1). 2/0 vicryl interrupted sutures were used to repair rupture of both corpus cavernosus. An 18-F Foley catheter was indwelled in the bladder (figure 2). The urethra was anastomosed in one layer, tension free with 5/0 PDS interrupted sutures (figure 3). Redundant foreskin was removed before reapproximation.

A broad spectrum antibiotic and low molecular heparin (dalteparin) were given during the hospital stay. Nocturnal erections recovered on the third postoperative day and they were mitigated with diazepam. On day 12 the catheter was removed and on day 13 the patient was released home. The antibiotic was continued at home for the next 10 days.

During the one year follow up period the patient presented with normal uroflowmetry one, six and twelve months after the surgery (max. flow rates were 22, 23 and 25 ml/s), with slight, stable and clinical insignificant urethral stricture on retrograde urethrogram 6 and 12 month after surgery and with normal voiding and sexual function.

## Discussion

Erection converts the safe, flaccid penis into a vulnerable organ. During erection thick tunica albuginea becomes thin and fracturable. Penile fracture is a relatively rare condition caused by a blunt trauma to the erected penis. The most frequent reported mechanism of trauma is unphysiological bending of the erect penis during sexual intercourse or masturbation [6].

Vigorous sexual intercourse is the main cause of penile fracture in the Western world. Because of high energy trauma urethral rupture is associated in up to 38% of penile fractures [7]. The majority of cases in the Eastern world are results of patients snapping and kneading of their penis during erection to achieve detumescence. Due to a low energy trauma the urethra is rarely involved. Zargooshi reported urethral rupture in 3% of penile trauma [8]. Usually urethral rupture is partial, rarely complete.

Early conservative treatment with cold applications, pressure dressings, catheterization, anti-inflammatory drugs, antibiotics and erection suppressing drugs is now replaced with immediate surgical repair. Surgical repair of penile fracture was first described by Fetter and Gartman in 1936 [9]. Since the repair reduces the complication of fracture it is now the gold standard for treatment of penile fractures [1,10].

We presented a case of a 37 year old man with penile fracture. Emergency surgical repair revealed bilateral partial rupture of the corpus cavernosum with complete urethral disruption. In one year follow up the patient presented with normal sexual and voiding function.

## Conclusion

Penile fracture is a rare urological condition. Emergency surgical repair can preserve voiding and sexual function.

## Competing interests

The author(s) declare that they have no competing interests.

## Authors' contributions

All authors actively participate by writing the article.
